# Gas gill respiration enhances underwater copulation in a water scorpion

**DOI:** 10.1016/j.isci.2026.117837

**Published:** 2026-10-13

**Authors:** Hyoseul Hyun, Nikolai J. Tatarnic, Chang S. Han

**Affiliations:** 1 Department of Biology, Kyung Hee University, Seoul 02447, Korea; 2 School of Biological Sciences, Monash University, Melbourne, VIC 3800, Australia; 3 Collections & Research, Western Australian Museum, Welshpool, WA 6106, Australia; 4 Centre for Evolutionary Biology, The University of Western Australia, Perth WA 6009, Australia; 5 Korea Institute of Ornithology, Kyung Hee University, Seoul 02447, Korea

**Keywords:** dissolved oxygen, underwater copulation, gas gill, respiration, water scorpion

## Abstract

To overcome physiological constraints during submergence, many air-breathing aquatic animals utilize an air store. If this store functions as a gas gill for dissolved oxygen uptake, this will extend underwater copulation when atmospheric oxygen is inaccessible. We tested this hypothesis using water scorpion *Nepa hoffmanni* (Hemiptera: Nepidae). Our field surveys showed that, although *N. hoffmanni* typically inhabit shallow water, males cannot access atmospheric oxygen via their respiratory siphon during underwater copulation. Laboratory experiments revealed active oxygen use from the air store beneath the wings during submergence. Furthermore, under higher dissolved oxygen treatments, males copulated longer and more frequently, while mate-guarding behavior remained unaffected. These findings provide the first direct evidence that dissolved oxygen uptake via a gas gill enhances copulation efficiency in air-breathing aquatic insects, highlighting the importance of environmental oxygen availability for aquatic reproductive strategies, especially in the context of ongoing climate-driven changes to freshwater ecosystems.

## Highlights

• Male water scorpions cannot breathe atmospheric air during underwater copulation
• Males rely on a subalar air bubble, which acts as a gas gill for oxygen uptake
• Low dissolved oxygen reduces copulation duration and frequency, but not guarding
• We provide the first evidence that the gas gill mechanism benefits insect mating

## Graphical abstract

**Figure:**
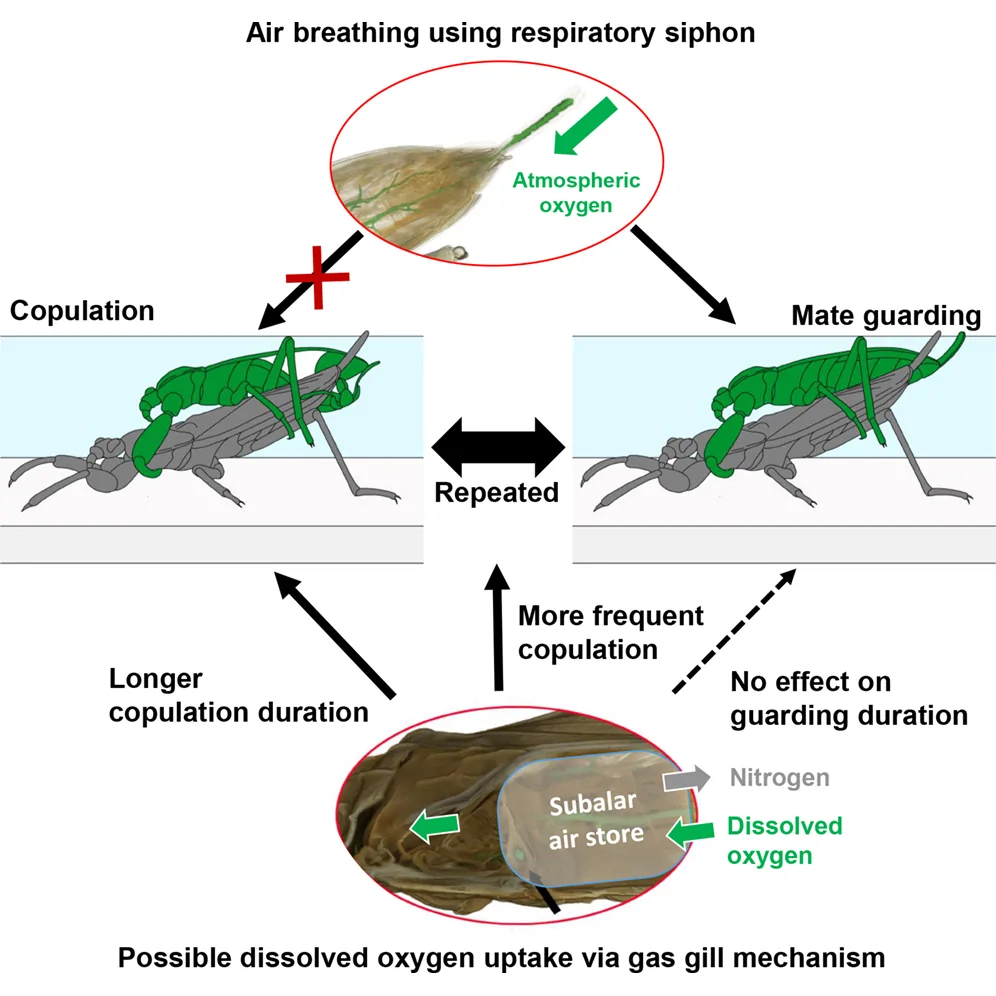

## Teaser

Zoology; Animal physiology; Animal morphology

## Introduction

During the evolutionary transition from an aquatic to a terrestrial lifestyle, many animals lost the ability to respire dissolved oxygen in water.^1^ Consequently, lineages that secondarily returned to aquatic habitats from terrestrial ancestors typically depend on atmospheric oxygen, forcing them to regularly surface and incur significant energy and foraging costs.^2^ To overcome these constraints, various air-breathing aquatic animals have re-evolved mechanisms for dissolved oxygen uptake, such as cutaneous respiration in certain reptiles and beetles.^3^^,^^4^

A more widespread strategy among air-breathing aquatic animals is the use of trapped air bubbles held by hydrophobic structures.^5^^,^^6^^,^^7^^,^^8^ These air bubbles act as physical gills or gas gills (hereafter referred to as a gas gill^8^^,^^9^^,^^10^), facilitating further dissolved oxygen uptake through diffusion.^10^ When a bubble forms underwater, the continuous consumption of oxygen from the bubble causes oxygen partial pressure within the bubble to decline, increasing the relative nitrogen partial pressure. This imbalance drives continuous gas exchange: dissolved oxygen diffuses into the bubble from the surrounding water, while nitrogen slowly diffuses out.^10^ Because nitrogen is progressively lost while oxygen is continually consumed, the bubble gradually shrinks. This process enables efficient dissolved oxygen uptake and prolong submergence.

Dissolved oxygen uptake confers major survival and reproductive benefits for air-breathing aquatic animals. For example, the water boatman *Hesperocorixa* sp. (Corixidae, Hemiptera) is a bottom-feeding aquatic insect that utilizes gas gills to remain underwater.^11^ However, under low-dissolved oxygen conditions, they frequently surface to breathe, increasing predation risk from surface-dwelling predators.^11^ Similarly, the backswimmer *Notonecta glauca* (Notonectidae, Hemiptera) relies on air bubbles to attack benthic prey more often when water is highly oxygenated.^12^ Moreover, dissolved oxygen uptake may enhance reproductive performance. For example, female water striders (*Aquarius paludum*) utilize air bubbles trapped on their body surface as gas gills to remain submerged and lay more eggs deeper underwater, avoiding parasitoids.^13^

While the physical mechanisms and ecological benefits of gas gills in foraging and survival are well documented, their role in sexual reproduction—specifically underwater copulation—remains largely unexplored. During underwater mating, males of air-breathing aquatic animals may be forced to surface for air before sperm transfer is complete. Such interruptions reduce opportunities for mate guarding and increase the risk of female insemination by rival males. If secondarily evolved respiratory adaptations such as gas gill allow males to remain submerged throughout copulation, uninterrupted copulation could substantially improve paternity. Yet, direct empirical evidence linking gas gill efficiency to copulatory success is still lacking.

In this study, we investigated the unaddressed reproductive benefits of gas gills in the water scorpion *Nepa hoffmanni* (Nepidae, Hemiptera) (Figure 1). Nepidae species breathe atmospheric air using a respiratory siphon at the tip of the abdomen—composed of two straps connected to the terminal abdominal spiracle—that provides continuous access to atmospheric air when its tip is held above the water surface.^14^^,^^15^ However, *N. hoffmanni* males are unable to use this siphon during copulation (Figure 1). While females keep their siphon intact and continue breathing air, males position their abdomen beneath the female’s genitalia, separating their siphon straps and submerging their abdominal spiracle (Figure 1, detailed in Hyun et al.^16^). This copulatory posture completely disables the siphon’s respiratory function during underwater copulation.

**Figure 1:**
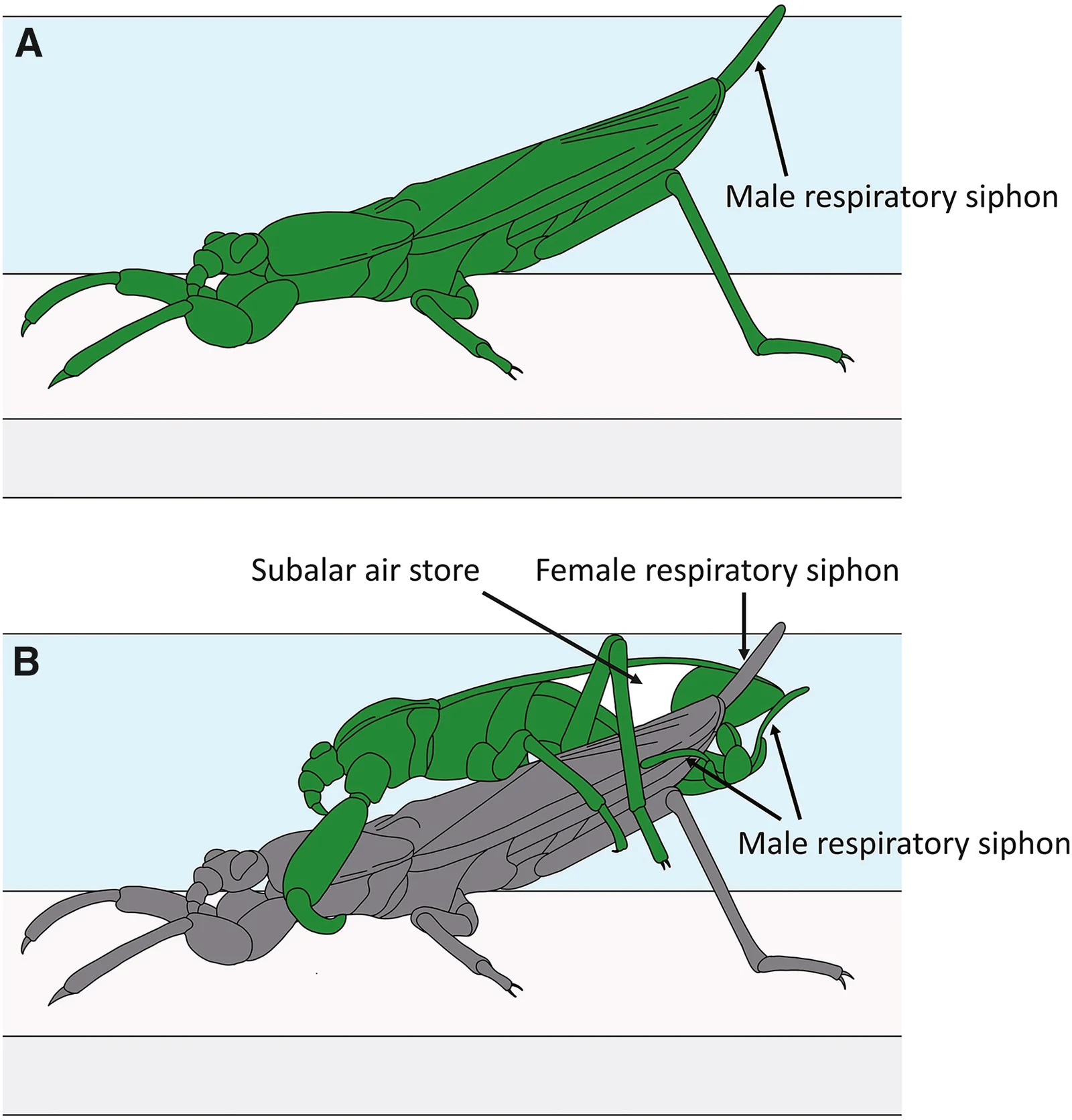
Schematic illustration of a single male and a male during copulation in *Nepa hoffmanni*; male and female individuals are colored green and gray, respectively (A) A male breathes by exposing its respiratory siphon above the water surface. (B) During copulation, the male twists his abdomen beneath the female’s genitalia, causing the respiratory siphon to separate. In this position, the divided siphon cannot function as an air passage; instead, the subalar air store (an air bubble located between the wings and abdomen) is exposed above the water. In contrast, the female retains an intact respiratory siphon during copulation, allowing continued access to air.

Nevertheless, *N. hoffmanni* is capable of acquiring oxygen while submerged. Nepidae species store air bubbles between the abdomen and wings (i.e., subalar air store^14^; Figure 1), which supports dissolved oxygen uptake. In *Nepa cinerea*, dissolved oxygen uptake via this store accounted for more than half of the total oxygen consumption when access to atmospheric air was restricted; however, this uptake decreased markedly when the subalar air store was prevented from contacting the surrounding water.^17^ Because male *N. hoffmanni* open a gap between their wings and abdomen during copulation (detailed in Hyun et al.^16^), we hypothesized that exposing this subalar air store to the surrounding water allows it act as an active gas gill (Figure 1). Thus, despite the loss of siphon function, males may rely on the subalar air store—as a static oxygen reserve or an active gas gill—to remain submerged and copulate longer.

To test this hypothesis, we combined micro-computed tomography (micro-CT) imaging, field surveys, and laboratory experiments. We first used micro-CT to identify the tracheal connections to the subalar air store and conducted field surveys to confirm that atmospheric oxygen is inaccessible during natural mating. Next, we monitored temporal changes in oxygen partial pressure within the subalar air store of both submerged living and dead specimens to confirm active oxygen consumption. Finally, we experimentally tested the dual role of the subalar air store as a static oxygen reserve versus as an active gas gill by comparing mating behaviors of pairs under different dissolved oxygen treatments. As *N. hoffmanni* exhibits repeated copulations while maintaining physical contact (copulation is followed by guarding and repeated several times, detailed in Hyun et al.^16^), we predicted that high dissolved oxygen conditions, where gas gills function more efficiently, would increase both copulation duration and frequency.

## Results and discussion

### Differences in water depth of single individuals versus pairs in the wild

In the wild, *N. hoffmanni* inhabits extremely shallow waters, allowing respiration via their respiratory siphon (Figure 1). Single males, single females, and mating pairs were found at mean water depths of 1.07, 1.43, and 1.82 cm, respectively (experiment 1; Figure 2). Among single individuals, females tended to inhabit at slightly greater depths than males (β, the estimated coefficient from linear [mixed-effects] models, (SE) = −0.46 (0.23), F_NUMdf, DENdf_ = 2.67_1,41.0_, *p* = 0.06, Figure 2). The water depths at which single individuals and mating pairs occurred did not differ significantly (β(SE) = 0.68 (0.42), F_NUMdf, DENdf_ = 3.15_1,53.0_, *p* = 0.12, Figure 2). However, one mating pair was observed at a depth of 9.5 cm, showing that copulation is not confined to the shallow water and can proceed where the siphon cannot readily reach the surface.

**Figure 2:**
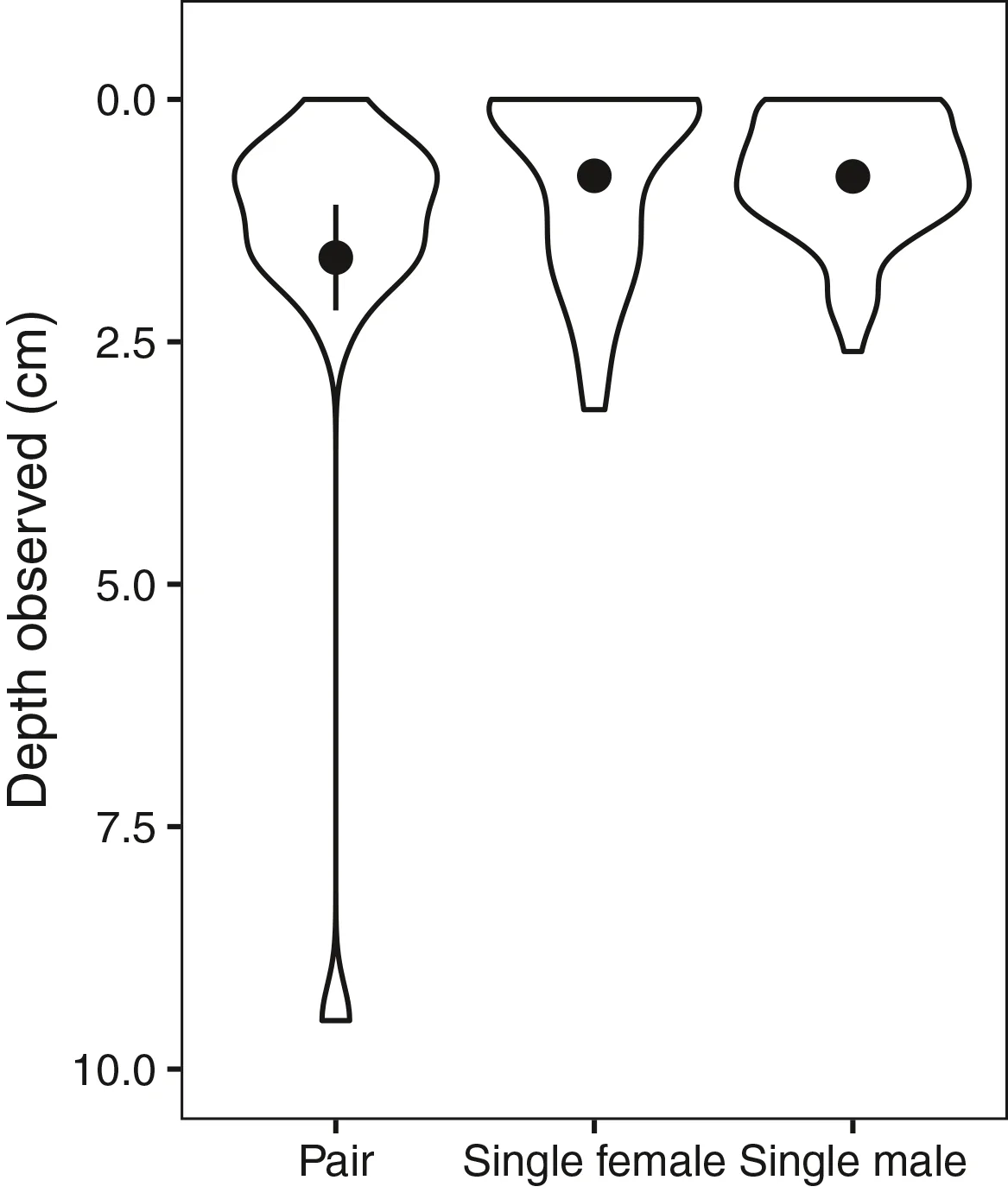
Water depths at which single males, single females, and pairs of *Nepa hoffmanni* were observed in wild Violin plots represent data distributions, while points and vertical lines indicate mean values and standard errors, respectively.

### The subalar air store as an alternative source of oxygen

However, water depth is largely irrelevant to the male’s respiratory capacity during copulation. Because the male’s copulatory posture separates the two-halves of the siphon, rendering it non-functional, male *N. hoffmanni* face a respiratory constraint during copulation irrespective of water depth. Our findings suggest that males overcome this challenge using the subalar air store. In Nepidae, most abdominal spiracles are non-functional; only the posterior spiracles (connected to the respiratory siphon) and the dorsal spiracles on the first abdominal segment, beneath the wing bases, remain functional.^14^^,^^18^ Indeed, micro-CT imaging confirmed these dorsal spiracles, which open into the air store beneath the wings (Figure 3). Consistent with active use of this store, the decrease in oxygen concentration within it over time was significantly greater in live than in dead individuals (experiment 2: β(SE) = 0.12 (0.004), F_NUMdf, DENdf_ = 901.50_1,588.0_, *p* < 0.001; Figure 4). After 10 min of submergence, oxygen concentration in live individuals had decreased by approximately 40% (from 18.31 to 10.96 kPa of oxygen concentration on average, SE = 1.75 kPa), whereas it remained nearly unchanged in dead individuals (from 18.69 to 18.49 kPa of oxygen concentration on average, SE = 0.01 kPa; Figure 4). Collectively, these findings indicate that *N. hoffmanni* can acquire oxygen from the subalar air store when the respiratory siphon is non-functional during underwater copulation.

**Figure 3:**
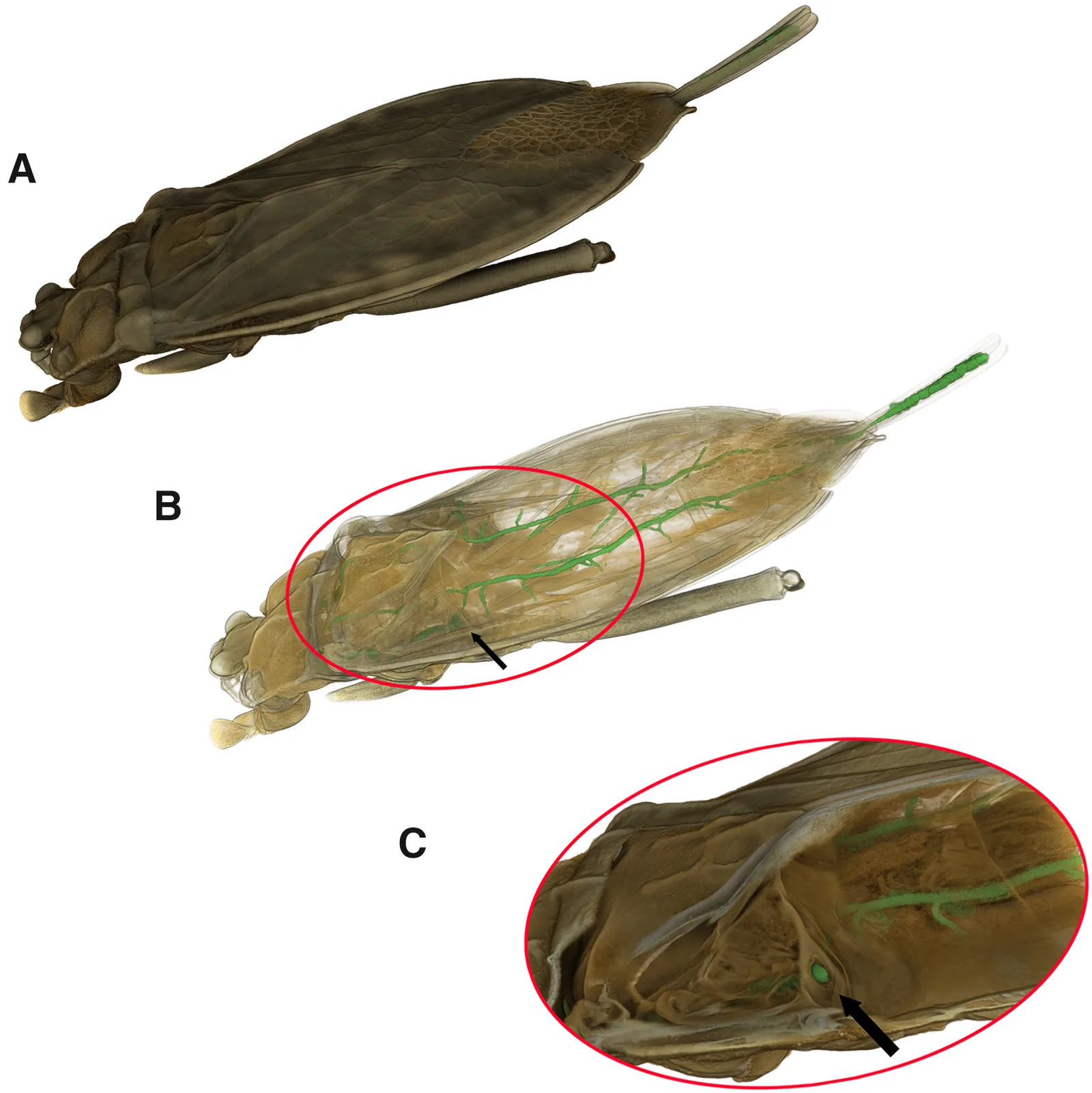
Micro-CT images of *Nepa hoffmanni* (A) The whole body; (B) the whole body with a transparent cuticle revealing the underlying tracheal structures (in green); and (C) a close-up of the circled area, with an arrow indicating one of the spiracles located dorsally on the first abdominal segment. This spiracle opens into the air store beneath the wings.

**Figure 4:**
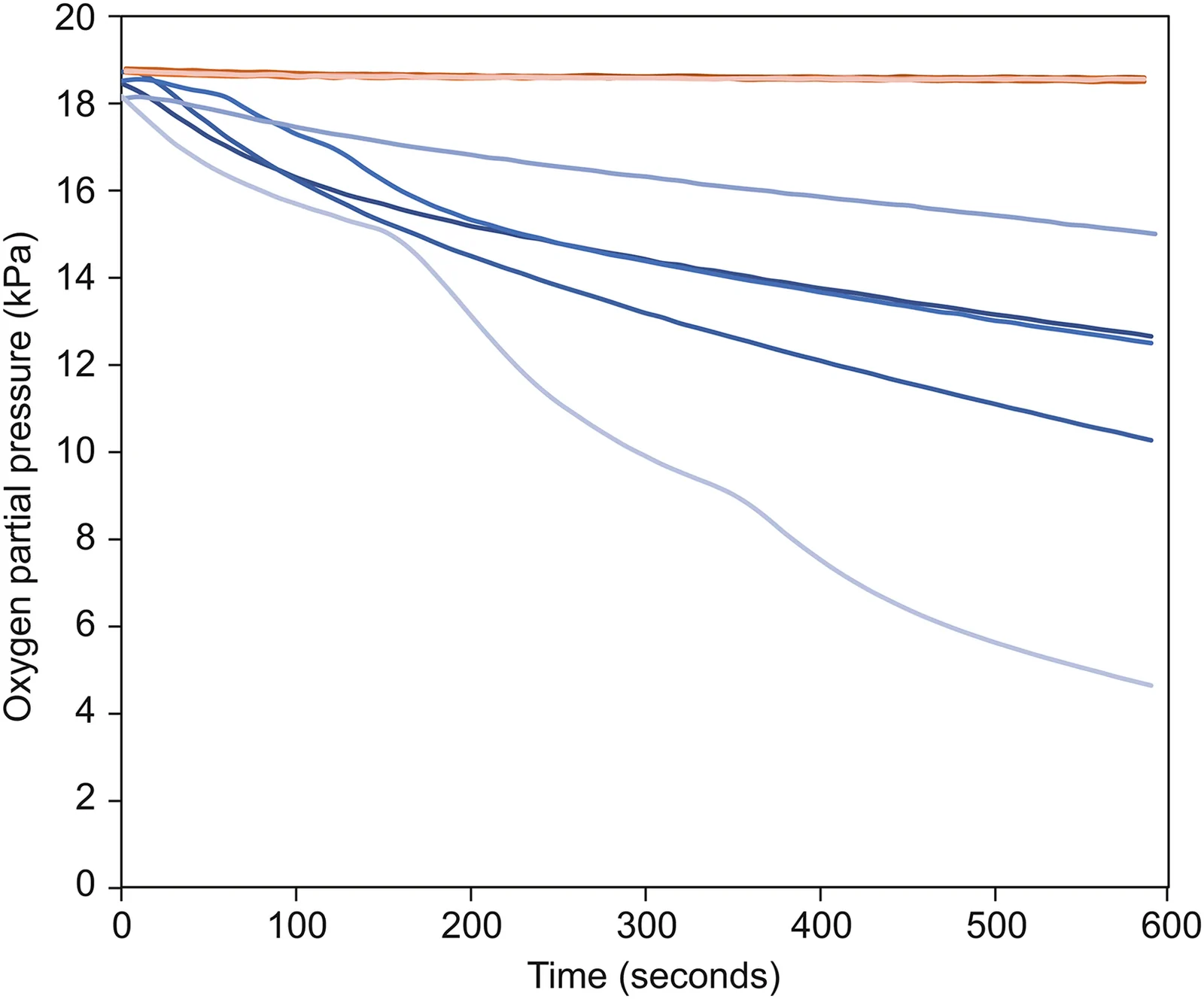
Oxygen partial pressure (kPa) within the subalar air store of *Nepa hoffmanni*, measured at 10 s intervals Blue and orange lines represent measurements from live (*n* = 5) and dead (*n* = 5) individuals, respectively.

### Effects of dissolved oxygen on copulation and mate guarding

While the air store could act merely as a static reservoir of oxygen trapped at the moment of submergence, males engaged in copulation likely face higher metabolic demands than resting males. We therefore asked whether they also actively extract dissolved oxygen from the surrounding water—with the subalar air store operating as a gas gill—to prolong submerged copulation (experiment 3). If the subalar air store functioned merely as a static reservoir—relying solely on the initial trapped oxygen without replenishment via a gas gill function— copulation duration and frequency would be expected to remain consistent regardless of ambient dissolved oxygen levels. In contrast to this null hypothesis, we found that *N. hoffmanni* copulation under high dissolved oxygen was longer per bout (single copulation duration, β(SE) = 1.88 (0.91), F_NUMdf, DENdf_ = 1.89_1, 32.4_, *p* = 0.02, Figure 5A) and more frequent (β(SE) = 3.00 (1.24), F_NUMdf, DENdf_ = 5.82_1,34.0_, *p* = 0.02, Figure 5C) than under low dissolved oxygen conditions (Figure 5). Consequently, total copulation duration was significantly greater under high dissolved oxygen conditions (β(SE) = 2149.83 (737.22), F_NUMdf, DENdf_ = 8.50_1,34.0_, *p* = 0.006, Figure 5B). Significant differences in copulatory performances between treatments persisted even after outlier removal (Results not shown). These results imply that when the efficiency of the gas gill is compromised by low ambient dissolved oxygen, males appear to deplete their energy reserves faster, resulting in shorter and fewer copulations. This pattern mirrors findings in aquatic gill-breathers, such as the Atlantic blue crabs *Callinectes sapidus*, which exhibits rapid leg fatigue under low dissolved oxygen.^19^ Thus, our findings provide evidence that oxygen uptake from the surrounding water is a limiting factor for sustaining underwater copulation in *N. hoffmanni*, supporting the functionality of the gas gill mechanism.

**Figure 5:**
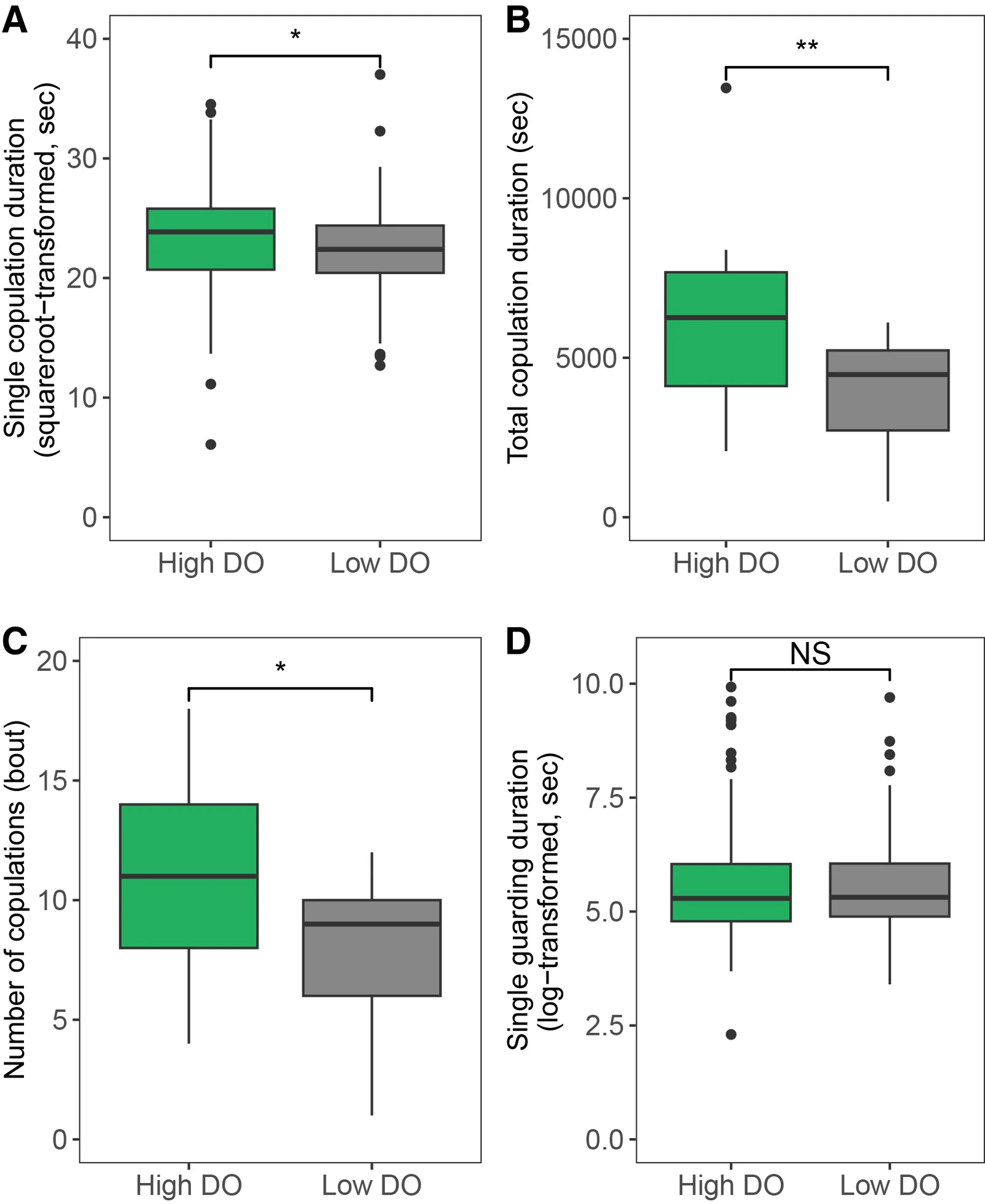
Effects of high versus low dissolved oxygen concentration on mating behaviors in *Nepa hoffmanni* (A) Single copulation duration, (B) total copulation duration, (C) number of copulations, and (D) single guarding duration. Boxplots show medians (solid lines) and interquartile ranges (25th and 75th percentiles). Whiskers (vertical lines) indicate data range excluding outliers, and black dots represent outliers. Asterisks denote significance levels for treatment effects (NS: *p* > 0.05; ∗: *p* < 0.05; ∗∗: *p* < 0.01).

Crucially, our analysis of mate guarding behavior serves as a robust control that strengthens this interpretation. Following copulation, males remain mounted on females but retract their genitalia and recombine their separated respiratory siphon.^16^ As a result, during mate-guarding, males can freely breathe atmospheric air underwater using their functioning respiratory siphon. If the reduced copulation performance under low dissolved oxygen were simply due to general metabolic depression caused by hypoxic water, one would expect guarding duration to be similarly affected. However, mate guarding duration remained consistent across dissolved oxygen treatments (β(SE) = −0.11 (0.26), F_NUMdf, DENdf_ = 0.61_1,30.4_, *p* = 0.44, Figure 5D). This contrast confirms that the sensitivity to dissolved oxygen is confined to the copulatory phase where the siphon is non-functional, reinforcing the gas gill mechanism.

### Reproductive and ecological implications of gas gill respiration

We suggest that dissolved oxygen uptake via the gas gill mechanism provides potential reproductive benefits to male *N. hoffmanni*. Although we did not measure sperm transfer directly, copulation duration typically correlates positively with sperm transfer quantity in many insect species.^20^^,^^21^^,^^22^^,^^23^^,^^24^ In addition, genital coupling itself can serve as a form of mate guarding.^25^^,^^26^^,^^27^

Taken together, our findings establish the following objective conclusions. We provide the first clear evidence that the gas gill mechanism provides mating advantages to air-breathing aquatic insects. Our results demonstrate that ambient dissolved oxygen levels strictly limit the duration and frequency of underwater copulation in *N. hoffmanni*, extending previous insights derived from gill-breathing animals.^28^^,^^29^^,^^30^ Finally, these findings highlight that reproductive strategies in aquatic animals are highly dependent on environmental oxygen availability, demonstrating the vulnerability of such reproductive performances to climate-driven hypoxia in freshwater ecosystems.

### Limitations of the study

We cannot entirely exclude the possibility that the observed differences in copulation duration and number of copulations across dissolved oxygen treatments might involve female responses. However, any female contribution is likely minimal, given that female underwater respiration during copulation was not physiologically constrained by the experimental dissolved oxygen levels. Unlike males, females can maintain unrestricted atmospheric respiration via their functional respiratory siphon throughout copulation. Furthermore, copulation in *N. hoffmanni* appears to be predominantly male-controlled; In both treatments, females exhibited no apparent resistance during mating, and mating was terminated by the voluntary dismounting of males, consistent with our previous description of this species’ mating behavior.^16^ Therefore, we argue that observed differences in reproductive performances under varying dissolved oxygen treatments primarily reflect male respiratory constraints. Nonetheless, caution is needed in interpreting these results because we could not control for age or mating history of experimental individuals. Due to their long lifespan of up to 3 years,^31^ it was impossible to ascertain the exact age or prior mating experience of field-collected individuals.

Additionally, although our experimental design contrasted conditions with and without gas gill function, a few limitations remain. First, we acknowledge that our high dissolved oxygen treatment provided mildly hyperoxic conditions. However, we argue that this elevated oxygen level is unlikely to have critically altered mating behavior of *N. hoffmanni*. A detailed justification for this experimental condition is provided in the STAR Methods. Second, we were unable to directly track the physical diffusion of dissolved oxygen molecules into the male’s air bubble during copulation. Future studies using radioactive oxygen isotopes would provide definitive, direct evidence of active gas gill function during underwater copulation of *N. hoffmanni*.

## Resource availability

### Lead contact

Request for more information and resources should be directed to the lead contact, Chang S. Han (hcspol@gmail.com).

### Materials availability

This study did not generate new materials.

### Data and code availability

• All data supporting this study are publicly available at https://doi.org/10.6084/m9.figshare.30519245.
• All statistical codes supporting this study are publicly available at https://doi.org/10.6084/m9.figshare.30519245.
• Any additional information required to reanalyze the data reported in this paper is available from the lead contact upon request.

## Acknowledgments

We gratefully acknowledge four anonymous reviewers for useful comments on earlier versions of the manuscript. We also acknowledge the facilities and the scientific and technical assistance of Microscopy Australia at the Center for Microscopy, characterization and analysis (CMCA), the University of Western Australia, a facility funded by the University, State and Commonwealth Governments. This work was supported by 10.13039/501100003725National Research Foundation of Korea grants funded by the Korean government (NRF-2022R1C1C1004303, RS-2025-16067311, RS-2025-25442355, RS-2026-25589531, and RS-2024-00405751) to C.S.H.

## Author contributions

H.H. and C.S.H. conceived and designed the study; H.H. collected the data; H.H. and C.S.H. analyzed the data; N.J.T. carried out the micro-CT scanning and analysis; H.H. and C.S.H. led the writing of the manuscript. All authors contributed critically to the drafts and gave their final approval for publication.

## Declaration of interests

The authors declare no competing interests.

## STAR★Methods

### Key resources table

**Table:** 

REAGENT or RESOURCE	SOURCE	IDENTIFIER
**Chemicals, peptides, and recombinant proteins**		

Nitrogen gas	Seoul Specialty Gases co.	CAS: 7727-37-9
Oxygen gas	Seoul Specialty Gases co.	CAS: 7782-44-7
Sodium Sulfite	Pyroscience	CAS: 7757-83-7

**Experimental models: Organisms/strains**		

*Nepa hoffmanni*	Gangneung-si, Gangwon-do, Republic of Korea	N/A

**Deposited data and code**		

Data and original code	This paper	https://doi.org/10.6084/m9.figshare.30519245

**Software and algorithms**		

Zeiss Versa 520 XRM imaging system	University of Western Australia’s Center for Microscopy and Microanalysis	https://www.zeiss.com/microscopy/en/products/x-ray-microscopy/versaxrm.html
Drishti2.6.5.	Limaye^32^	https://doi.org/10.1117/12.935640
ASReml 4.2	VSN Interaction	https://vsni.co.uk/software/asreml-sa/

**Other**		

Firesting-G02	Pyroscience	https://www.pyroscience.com/en/products/all-meters/fsgo2

### Experimental model and study participant details

#### Study species

*Nepa hoffmanni* is an aquatic insect widely distributed in East Asia, including Korea, China, Japan, and the eastern regions of Russia.^31^ They terminate winter reproductive diapause in March and remain reproductively active thereafter, mating multiple times throughout the reproductive period.^31^ However, it remains unclear whether the summer generation is also reproductively active. *N. hoffmanni* typically inhabits shallow creeks and pools adjacent to mountain streams.^31^ Mating occurs underwater, with detailed mating behaviors described by Hyun et al.^16^

#### Sample collection and maintenance

For experiments 2 and 3, we collected *N. hoffmanni* from streams and springs in Gangneung-si, Gangwon-do, between March and April 2023. Each individual was housed in an acrylic home container (diameter: 10 cm; height: 4 cm), filled with 1 cm of tap water and provided with styrofoam pieces as refuge. Crickets (*Gryllus bimaculatus*) were supplied as food every three days, and water was replaced one day after feeding. Both rearing and laboratory experiments (experiments 2 and 3) were conducted in a controlled room maintained at 23°C with a 17:7-h light-dark cycle.

### Method details

#### Micro-CT imaging

X-ray microtomography (micro-CT) was conducted at the University of Western Australia’s Center for Microscopy and Microanalysis (CMCA), using a Zeiss Versa 520 XRM imaging system. As tracheal systems are not sexually dimorphic, a female specimen preserved in 100% ethanol was first stained in I2E (1% iodine by weight dissolved in 100% ethanol) for 24 hours to enhance contrast.^33^ The specimen was then rinsed in ethanol, then sealed in a 100% ethanol-filled 5 mL plastic vial. This was then mounted in the micro-CT for scanning. Two datasets were generated, one of the whole body (comprising three sequential scans vertically stitched together during reconstruction), and a second at higher resolution of only the thorax. For both datasets the source was set at 80 kV, 7W with an LE1 filter, with 2001 image at 1.2 seconds exposure taken as the specimen was rotated through 360°. The whole-body dataset was generated using a 0.4X scintillator objective lens, resulting in a voxel resolution of 13.527 μm, while the thorax scan used the 4X scintillator lens, resulting in a resolution of 10.775μm. Micro-CT datasets were automatically reconstructed using default settings in XMReconstructor (v11.1.5707.17179). Visualisation and imaging were then conducted using Drishti2.6.5.^32^ Although the tracheoles themselves were not directly captured by the X-rays, the negative space within could be observed and tagged in the Drishti Paint package by manipulating the transfer function to visualise those voxels representing little or no X-ray absorption (i.e., empty space and ethanol). The negative space within the tracheoles could then be manually tagged and extracted, then visualized in Drishti alongside the tagged tissues of the animal.

#### Experiment 1. Differences in water depth of single individuals versus pairs in the wild

To investigate the respiratory conditions in the natural habitat of *N*. *hoffmanni*, we recorded the water depths at which single males, single females, and mating pairs were found. Observations were conducted at three sampling sites in Gangneung-si, Gangwon-do, between 31 March and 3 April 2023, after sunset when *N. hoffmanni* becomes active. For each observation, we recorded the sex (male or female), pairing status (single or paired), and the water depth and temperature at the individual’s location.

#### Experiment 2. Temporal changes in oxygen partial pressure in the subalar air store of live versus dead individuals

To test whether *N. hoffmanni* uses its subalar air store for respiration, we monitored temporal changes in oxygen concentration within the subalar air store in 5 live and 5 dead individuals using a fiber-optic oxygen sensor (Pyroscience Firesting-G02). A 200 μm pipette tip was attached to the sensor, with moistened cotton was wrapped around it to prevent air exchange with the surroundings. The gap between the abdomen and wings was gently opened by compressing the abdomenal sides with forceps, and the pipette tip was inserted into the subalar air store. Each insect was submerged entirely, including its respiratory siphon, in a 50 mL vial filled with tap water at 22°C, with the head positioned at the bottom. This arrangement allowed the subalar air store to connect with air trapped in the pipette tip (Figure S1). We measured oxygen concentration in the subalar air store for 10 minutes at 10-s intervals.

#### Experiment 3. Differences in mating behavior under low and high oxygen treatments

To assess the effect of dissolved oxygen on the mating behavior of *N. hoffmanni*, we prepared two water treatments differing in dissolved oxygen concentration. “High dissolved oxygen “ water was created by infusing 99% oxygen gas into 23°C tap water, maintaining a consistent dissolved oxygen concentration of approximately 25.76kPa (at 23°C and 1atm). “Low dissolved oxygen” water was prepared by replacing the dissolved oxygen in 23°C tap water with 99.99% nitrogen gas, consistently maintaining a dissolved oxygen concentration of around 5.15kPa (at 23°C and 1atm). Dissolved oxygen concentrations were monitored using a fibre-optic oxygen sensor (Pyroscience Firesting-G02). Mating behavior was recorded in a transparent acrylic chamber containing 20 arenas (internal dimensions: 6cm x 6cm x 3cm), arranged in 5 rows and 4 columns. Each arena had a transparent acrylic stick (3 mm × 3 mm × 10 mm) vertically attached at the center of the base to allow insects to maintain stable postures underwater (Figure 6). Each arena was filled with 1.5 cm of water and covered with an aluminum mesh (6 cm × 6 cm). This mesh held both the male and female submerged during mating, allowing air-breathing via respiratory siphons for females throughout the experiment but for males only during the guarding period (Figures 6A, S2A, and S2B). During copulation, males were prevented from accessing atmospheric air (Figures 6B, S2C, and S2D).

**Figure 6:**
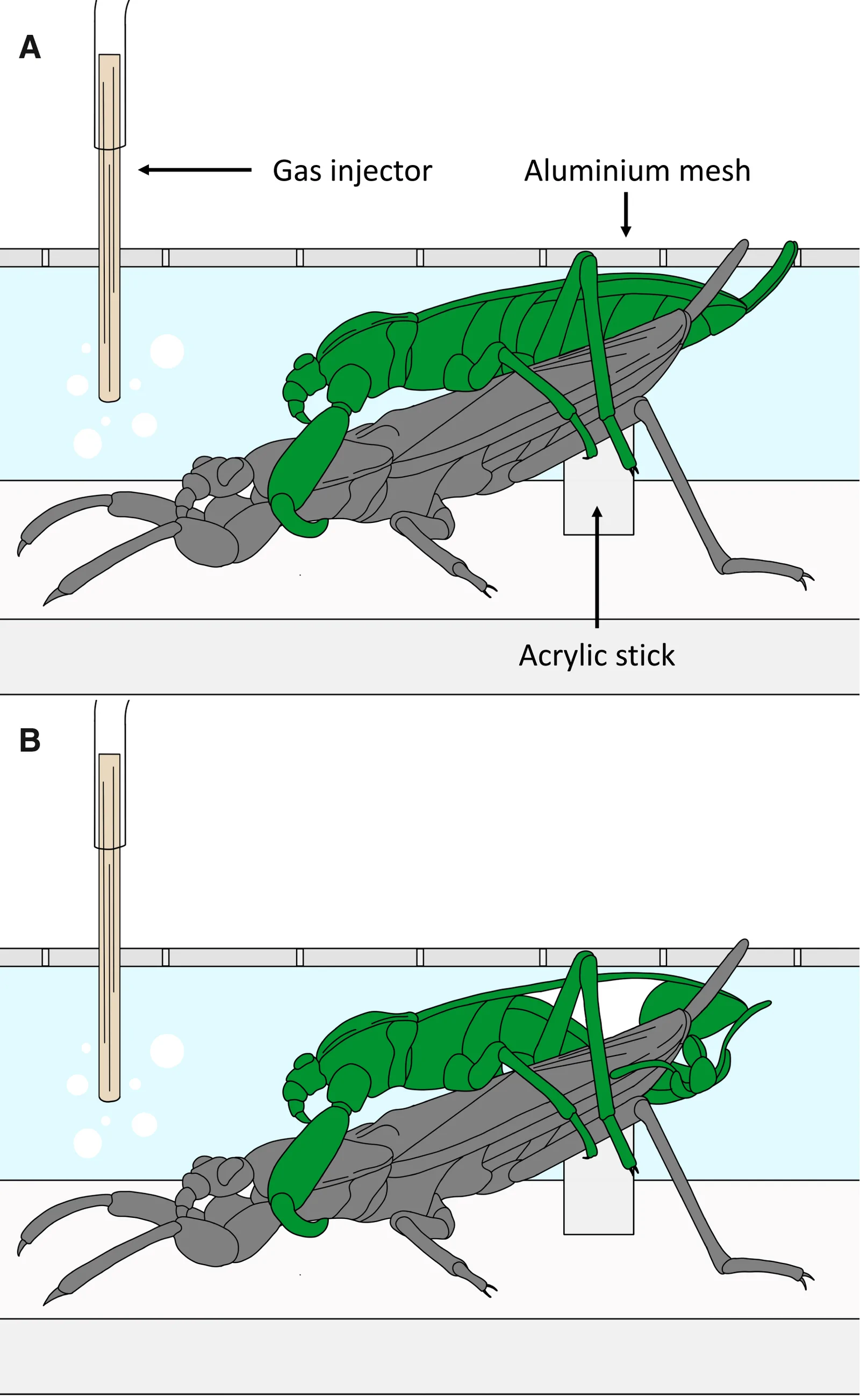
Schematic illustration of a mating pair of *Nepa hoffmanni* during guarding and copulation under the experimental setup used in experiment 3 Male and female individuals are colored green and gray, respectively. (A) During guarding, both individuals can expose their respiratory siphons above the water surface, which is covered by aluminum mesh. (B) During copulation, the male’s respiratory siphon separates, preventing him from exposing his abdominal tip above the water surface for air breathing.

To maintain stable dissolved oxygen concentrations, oxygen or nitrogen gas was continuously supplied into each arena using injectors connected to the respective gas tanks. Each injector comprised a 3 mm diameter tube with a 35 mm bamboo stick tip to disperse fine bubbles without disturbing mating behavior. Because oxygen dissolves more readily than nitrogen, gas injectors in the high dissolved oxygen chamber were connected to both an oxygen tank and an air pump; a dissolved oxygen sensor placed in one arena was used to monitor and adjust dissolved oxygen levels as required. Throughout the experiment, we continuously monitored dissolved oxygen concentration, temperature, and atmospheric pressure using a fibre-optic oxygen sensor.

For the mating assay, a single male and female were transferred from their home containers into an experimental arena. To minimise potential variation arising from the unknown age and mating history of field-collected individuals, all individuals underwent a uniform acclimation period under controlled environmental conditions (i.e., the same climate room). Each experimental animal was individually isolated for one week prior to the assay to standardise their reproductive status and mitigate the influence of prior mating experiences in the wild. We then observed mating behaviors of *N.hoffmanni* pairs for up to 8 hours from a ventral perspective. During each mating event (from initial interaction to pair separation), we recorded five mating behaviors:

(1) Single copulation duration: time from successful intromission to withdrawal of the male genitalia
(2) Total copulation duration: sum of single copulation durations across repeated copulations
(3) Single guarding duration: time from genitalia withdrawal to male genitalia extension for the next copulation
(4) Number of copulations: total number of copulations within a mating event (from initial interaction to pair separation)

Detailed descriptions of these mating behaviors are provided in Hyun et al.^16^

#### “High” and “Low” dissolved oxygen conditions for experiment 3

To test whether the gas gill affects mating efficiency, we manipulated dissolved oxygen levels to effectively enhance or suppress the gas exchange capacity of the subalar air store throughout the experiment. We established two distinct experimental conditions: a “High dissolved oxygen” treatment (oxygen-enriched) and a “Low dissolved oxygen” treatment (nitrogen-enriched).

To ensure accurate measurements, the oxygen sensor was calibrated within the same temperature-controlled climate room used for the experiments. A 0% saturation point was established using a sodium sulfite solution as an anoxic standard. Furthermore, to ensure precise temperature compensation, the FireSting-GO2 device utilised a connected external temperature probe in addition to its internal temperature sensor. This setup allowed us to monitor both water and ambient air temperatures simultaneously, ensuring valid automatic temperature compensation during both calibration and experimental procedures.

For the Low dissolved oxygen treatment, we targeted a hypoxic concentration of approximately 5.15 kPa (at 23°C and 1atm) by bubbling the water with 99.99% nitrogen gas, which was continuously injected throughout the experiment. To verify that the low dissolved oxygen concentration was consistently maintained over time, we conducted preliminary monitoring for 50 minutes under experimental conditions. These data confirmed that the Low dissolved oxygen treatment effectively maintained a stable hypoxic environment with a mean dissolved oxygen concentration of 6.57kPa (SD = 0.76) at 24.82 °C (SD = 0.62), corresponding to approximately 32% saturation (Table S1).

For the High dissolved oxygen treatment, we aimed to establish a dissolved oxygen concentration of 25.76 kPa (at 23 °C and 1atm) by saturating the water with 99% oxygen gas. This concentration was selected to reflect the average dissolved oxygen concentrations (24.78 – 26.70kPa) measured in 2020 across five major water supply sources near Seoul, Korea. We intentionally established mild hyperoxia conditions to ensure that the partial pressure of oxygen in the water remained consistently higher than that within the subalar air store of *N. hoffmanni*.

Although this condition represents mild hyperoxia relative to standard air saturation, it is ecologically relevant. In natural aquatic habitats—particularly the shallow waters inhabited by *N. hoffmanni*—photosynthetic activity can frequently induce hyperoxic conditions during peak daylight hours (reviewed in McArley et al.^34^). Furthermore, moderate hyperoxia is generally reported to have negligible impacts on the physiology of terrestrial air-breathing insects (reviewed in Harrison et al.^35^). Similarly, in aquatic air-breathing insects, survivorship at critical thermal maxima is either unaffected or slightly enhanced under the 3-fold greater dissolved oxygen tension than normal condition.^36^^,^^37^ Therefore, we argue that the mild hyperoxia in our High dissolved oxygen treatment is unlikely to alter mating behavior or generate artifacts distinct from natural condition. However, due to the higher solubility of oxygen compared to nitrogen, continuous infusion of pure oxygen quickly increased the oxygen concentration beyond 51.52 kPa (at 23 °C and 1atm). To prevent excessively high dissolved oxygen levels, we connected the gas injector to both a 99% oxygen gas tank and an air pump. Preliminary monitoring for 30 minutes confirmed that this setup effectively maintained a stable mean dissolved oxygen concentration of 25.90kPa (SD = 1.99) at 23.34 °C (SD = 0.07), equivalent to approximately 127% saturation (Table S1).

### Quantification and statistical analysis

#### Statistical analysis

##### Experiment 1

To assess sex differences in the water depth where individuals were found in the wild, we employed a linear mixed-effects model with water depth as the response variable, assuming Gaussian error. The model included sex (male vs female), water temperature and observation time as fixed effects. Sampling location was included as a random effect to account for spatial clustering. In addition, to assess differences in water depth between single individuals and pairs, we employed another linear mixed-effects model with water depth as the response variable. This model included pairing status (single vs pair) as a fixed factor and incorporated the same covariates and random factor as the previous model. For both models, assumptions of residual normality were verified via visual inspection of residual plots.

##### Experiment 2

To assess differences in the decline of oxygen concentration within the subalar air store between live and dead individuals, we employed a linear mixed-effects model assuming a Gaussian error distribution. The response variable was the change in oxygen concentration from the initial measurement. The model included treatment (live vs dead), time unit (1 unit = 10 seconds), and their interaction as fixed effects. Individual ID was included as a random effect to account for the non-independence of repeated measures collected from the same individual. Model assumptions of residual normality were again confirmed via visual inspection of residual plots.

##### Experiment 3

To assess differences in mating behavior under low and high dissolved oxygen treatments, we built univariate linear (mixed-effects) models. Single copulation duration was square root-transformed, while single guarding duration was log-transformed to normalise residuals, as confirmed visually. In models with single copulation duration or single guarding duration as a response variable, dissolved oxygen treatment (low vs. high), number of copulations, and their interaction were included as fixed effects. Male ID was included as a random effect to account for the non-independence of repeated measures collected from the same mating pair across repeated copulations. In models with total copulation duration or number of copulations as a response variable, dissolved oxygen treatment was included as the only fixed effect because each pair provided a single value of those behaviors.

We assessed the significance of fixed effects and covariates using Wald F-tests and the significance of random effects using likelihood ratio tests (LRTs). The test statistic for the LRTs was calculated as twice the difference in log likelihood between models with and without a random effect. The *p* value for the random effect was then derived, assuming a mixture distribution of P (χ2, df = 0) and P (χ^2^, df = 1).^38^^,^^39^^,^^40^ These results are presented as “χ^2^_0/1_”. All the statistical analyses were performed in ASReml 4.2 (VSN Interaction, Hemel Hempstead, UK) and solved by restricted maximum likelihood.^41^
