# Supplementary material for: Gas gill respiration enhances underwater copulation in a water scorpion
Source: iScience. 2026 Oct 13;29(11):117837. doi: 10.1016/j.isci.2026.117837 (PMC13683752; doi:10.1016/j.isci.2026.117837)
Supplement: Supplementary file 1 [file mmc1.pdf]

iScience, Volume *29*

Supplemental information

Gas gill respiration enhances underwater copulation in a water scorpion

Hyoseul Hyun, Nikolai J. Tatarnic, and Chang S. Han

Supplementary information

Figure S1. Photographs illustrating the experimental setup for experiment 2. The left probe measures water temperature, while the right probe—inserted into a pipette tip—records the oxygen concentration (%) within the subalar air store of *Nepa hoffmanni*. Both vials were filled with tap water and acclimatised under identical experimental conditions.

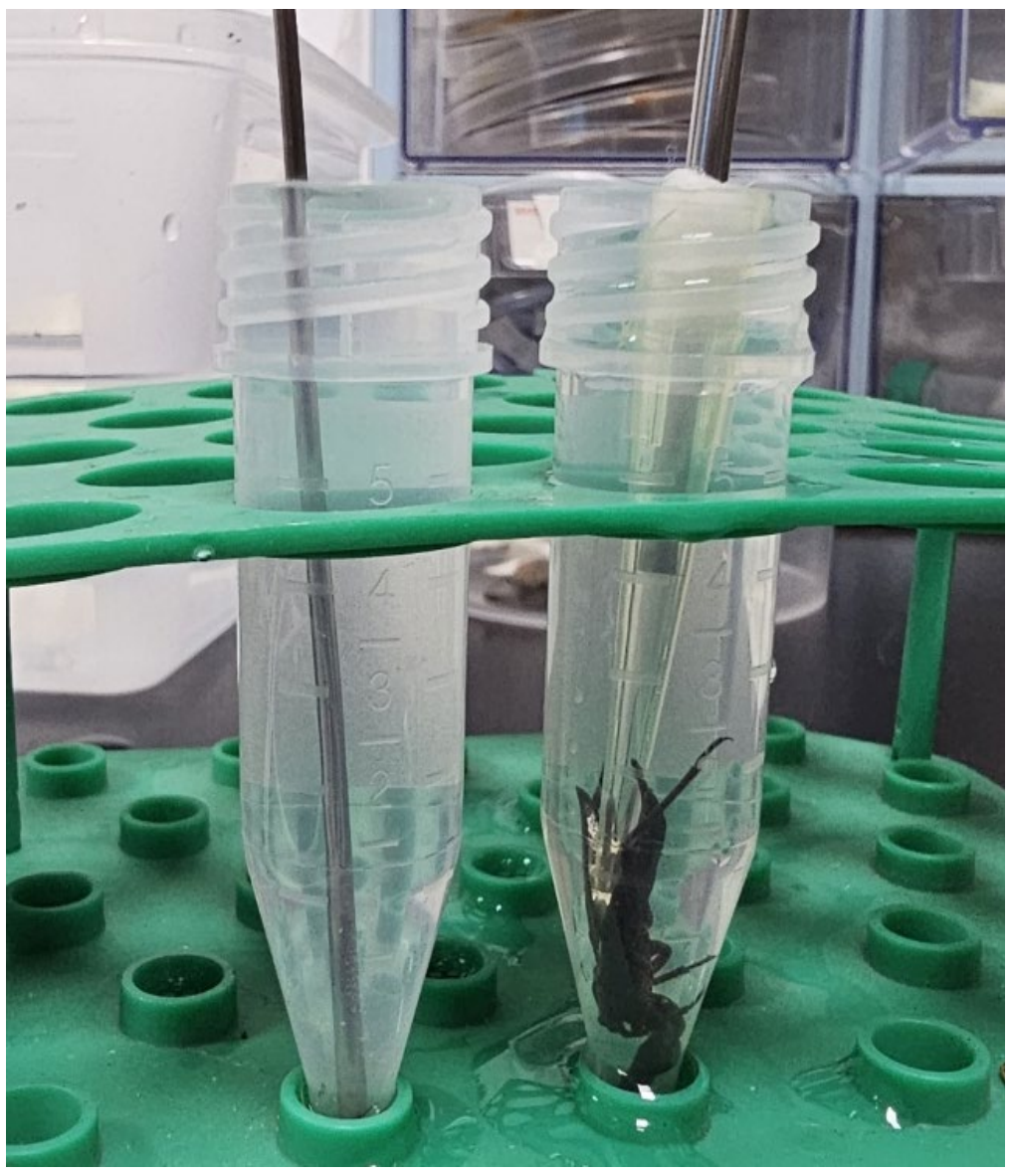

Figure S2. Photographs of *Nepa hoffmanni* mating pairs from experiment 3. (A, B) Dorsal and ventral views of a mating pair during guarding. (C, D) Lateral and ventral views of a mating pair during copulation.

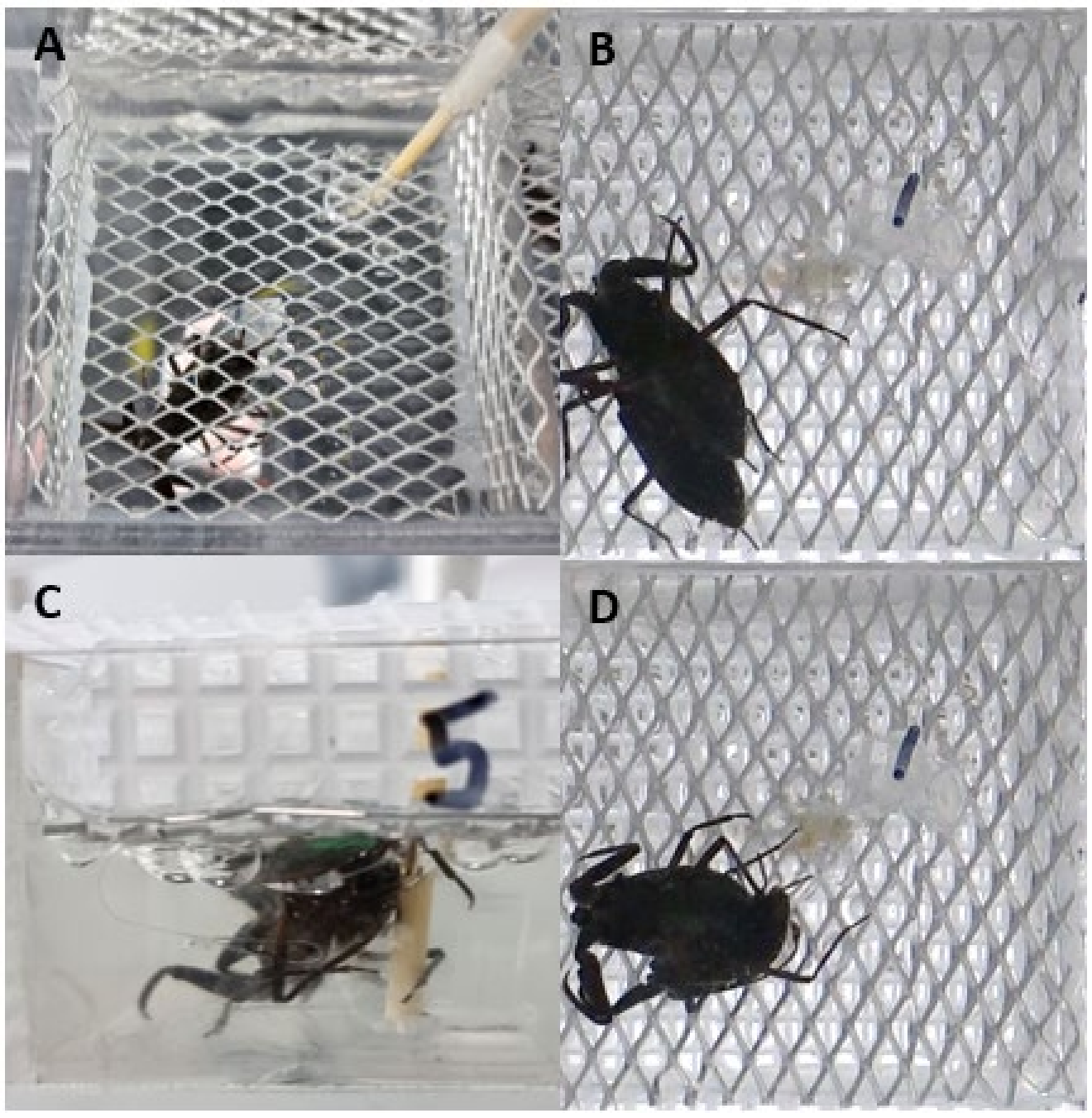

Table S1. Monitoring of dissolved oxygen concentrations under “Low” and “High” dissolved oxygen treatments in the experiment. Partial pressure (kPa) and percentage (%) saturation values were calculated from the monitoring data following guidelines from Mortimer [1] and the PyroScience manual.

	Unit	Low DO	High DO
Average DO concentration	kPa (SD)	6.57 (0.78)	25.90 (1.99)
	% saturation	32	127
Minimum DO concentration	kPa	5.43	23.02
	% saturation	26	113
Maximum DO concentration	kPa	7.51	30.52
	% saturation	36	149
Average temperature	°C (SD)	24.82 (0.62)	23.34 (0.07)
Average air pressure	atm	1.01	0.99

References

1. Mortimer, C.H. (1981). The oxygen content of air-saturated fresh waters over ranges of temperature and atmospheric pressure of limnological interest. Internationale Vereinigung für Theoretische und Angewandte Limnologie: Mitteilungen *22*, 1-23. https://doi.org/10.1080/05384680.1981.11904000.
